# Cytotoxic and targeted therapy for *BRCA1/2*-driven cancers

**DOI:** 10.1186/s13053-021-00193-y

**Published:** 2021-08-28

**Authors:** Evgeny N. Imyanitov

**Affiliations:** 1 N.N. Petrov Institute of Oncology, Pesochny, Saint-Petersburg, 197758 Russia; 2 St.-Petersburg Pediatric Medical University, Saint Petersburg, 194100 Russia; 3 I.I. Mechnikov North-Western Medical University, St.-Petersburg, 191015 Russia

**Keywords:** *BRCA1*, *BRCA2*, Cisplatin, Carboplatin, Mitomycin C, PARP inhibitors, BRCAness

## Abstract

Tumors arising in *BRCA1/2* germline mutation carriers usually demonstrate somatic loss of the remaining *BRCA1/2* allele and increased sensitivity to platinum compounds, anthracyclines, mitomycin C and poly (ADP-ribose) polymerase inhibitors (PARPi). Exposure to conventional platinum-based therapy or PARPi results in the restoration of *BRCA1/2* function and development of resistance to systemic therapy, therefore, there is a need for other treatment options. Some studies suggested that the use of specific drug combinations or administration of high-dose chemotherapy may result in pronounced tumor responses. *BRCA1/2*-driven tumors are characterized by increased immunogenicity; promising efficacy of immune therapy has been demonstrated in a number of preclinical and clinical investigations. There are outstanding issues, which require further consideration. Platinum compounds and PARPi have very similar mode of antitumor action and are likely to render cross-resistance to each other, so their optimal position in cancer treatment schemes may be a subject of additional studies. Sporadic tumors with somatically acquired inactivation of *BRCA1/2* or related genes resemble hereditary neoplasms with regard to the spectrum of drug sensitivity; the development of user-friendly BRCAness tests presents a challenge. Many therapeutic decisions are now based on the *BRCA1/2* status, so the significant reduction of the turn-around time for predictive laboratory assays is of particular importance.

## Introduction

The development of tumors in *BRCA1/2* germ-line mutation carriers usually includes somatic inactivation of the remaining allele of the involved gene. Consequently, these malignancies are characterized by tumor-selective BRCA1/2 deficiency, down-regulation of DNA double-strand break repair and high-level chromosomal instability. These features of *BRCA1/2*-driven cancers underlie their specific pattern of sensitivity to cytotoxic and targeted compounds. This review discusses the latest developments in the therapy of *BRCA1/2*-associated malignancies.

## Cytotoxic therapy

Platinum-based cytotoxic drugs form DNA crosslinks, which are believed to ultimately cause DNA double-strand breaks and activate DNA repair by homologous recombination. Recent studies updated this concept indicating that other mechanisms, i.e., the formation of single-stranded DNA replication gaps, may underlie an increased sensitivity of BRCA1/2-deficient cells to cisplatin or carboplatin [1]. Clinical validation of these data present a challenge. Platinum salts form a backbone for the therapy of ovarian cancer (OC); however, these agents are almost always given in combination with other drugs, with carboplatin/paclitaxel being the most common regimen in the past. Retrospective and prospective studies revealed that *BRCA1/2* mutation carriers obtain more benefit from a standard therapy for OC as compared to women with non-hereditary OC disease [2–4]. Investigations involving breast cancer (BC) patients are more complicated, given that platinum-containing schemes were not included in the standard BC treatment options until recently. Consequently, it took a few interventional trials to reveal that the replacement of the standard chemotherapy by single-agent cisplatin is indeed a promising option for clinical management of *BRCA1*-driven BC [5–8]. However, there are at least two outstanding issues: 1) how the performance of platinum-based therapy compares with the efficacy of other drug regimens? 2) what is the best companion to be added to the platinum backbone?

There are only a few studies, which directly compared single-agent platinum vs. conventional non-platinum therapy in BC patients with germ-line *BRCA1/2* mutations. Byrski et al. [5] analyzed mainly patients with recurrent Slavic/Jewish mutations in *BRCA1* gene and revealed higher efficacy of neoadjuvant cisplatin as compared to retrospective data obtained with the use of standard neoadjuvant regimens. However, these observations were not replicated in a recent randomized study, which utilized doxorubicin/cyclophosphamide as a comparator to cisplatin and involved patients with diverse mutations and *BRCA1* and *BRCA2* genes [9]. For the time being, it is safe to conclude that single-agent cisplatin may be considered as an option for the neoadjuvant treatment of BC in *BRCA1/2* mutation carriers. However, although this drug frequently induces pathologic complete (pCR) responses, many platinum-treated women still present with a residual tumor mass at surgery [7, 8]. Therefore, it is feasible to examine whether the addition of other therapeutic agents to the cisplatin backbone will result in the improvement of the pCR rate in neoadjuvant BC setting. Single-agent carboplatin showed significantly better efficacy than docetaxel in *BRCA1/2* germ-line mutation carriers, which were analyzed as a subgroup within a randomized trial for patients with triple-negative advanced BC [10].

Several laboratory studies suggested sensitivity of BRCA-deficient cells to mitomycin C. These findings have been confirmed by clinical data. Single-agent mitomycin C demonstrated activity towards heavily pretreated OC patients with *BRCA1* mutations [11]. Combination of mitomycin C and cisplatin showed good performance both in neoadjuvant setting and in the treatment of recurrent OC disease in *BRCA1* mutation carriers, being clearly superior to the carboplatin/paclitaxel or other regimens [12–14]. Importantly, some OC patients exposed to mitomycin/cisplatin-containing neoadjuvant chemotherapy experience complete pathologic responses, while standard drug schemes almost never result in complete elimination of OC cells [12, 14]. These data call for the evaluation of mitomycin/cisplatin efficacy in BC patients carrying *BRCA1/2* germ-line mutation. There are reports suggesting that combination of cisplatin and anthracyclines may exert significant therapeutic activity in *BRCA1*-driven hereditary BCs [15]. Choice of companion to cisplatin may depend on the spectrum of drugs utilized in the standard treatment for a given cancer type. For example, cisplatin/gemcitabine doublet demonstrated a remarkable efficacy in *BRCA1/BRCA2/PALB2*-driven hereditary pancreatic cancer [16].

Two very recent studies demonstrated increased sensitivity of BRCA1/2-driven tumors to bifunctional alkylating agents. Bifunctional alkylating drugs are able to generate DNA crosslinks and in this respect resemble platinum compounds [17]. Melphalan showed remarkable activity in women with recurrent hereditary BRCA1/2-mutated OC, whose disease was classified as platinum-resistant according to the duration of platinum-free interval [18]. Patients with BRCA1/2-associated relapsed OC experienced significant benefit from metronomic oral cyclophosphamide [19]. In line with these data, there are reports describing successful utilization of metronomic cyclophosphamide as a maintenance therapy, i.e., in the setting which resembles the current use of PARP inhibitors [20].

There is a high number of investigations, which compared conventional multidrug regimens in various categories of BC patients, and analyzed the outcomes in subgroups of women with germ-line *BRCA1/2* mutations. It is generally agreed, that the use of anthracyclines produces good results in patients with *BRCA1/2*-driven tumors [5, 9, 21, 22]. Many current treatment schemes involve the use of taxanes, and the analysis taxane-containing regimens is highly complicated due to a number of important nuances. Presence of BRCA1 is essential for taxane-mediated apoptotic death; some although not all studies suggested that the inclusion of taxanes into the drug cocktail compromises the efficacy of chemotherapy for *BRCA1*-associated tumors [5, 21, 23]. This statement may not be applicable to *BRCA2*, as the latter gene is not essential for taxane-driven killing of cancer cells [24]. Virtually all available studies pooled together *BRCA1*- and *BRCA2*-mutated cancers; this approach may be acceptable for the evaluation of the treatment outcomes for *BRCA1/2*-specific agents (anthracyclines, platinum drugs, mitomycin C, PARP inhibitors), but is questionable while considering the analysis of efficacy of taxanes or some other drugs. Even more importantly, the mode of administration of taxanes may play a role in the treatment outcomes, at least in theory. Some schemes utilized concurrent administration of taxanes with other cytotoxic agents, while it is also common to practice sequential use of anthracyclines and microtubule inhibitors [22, 23, 25–27]. If we speculate, that the treatment by anthracyclines results in the restoration of BRCA1 function via secondary mutation or other mechanisms [24, 28], the subsequent use of taxanes is likely to render the benefit similar to the one observed in non-selected BC patients.

The distinction between *BRCA1* and *BRCA2* may indeed be of importance in some circumstances. *BRCA2*-associated tumors demonstrate higher sensitivity to trabectedin or lurbinectedin than *BRCA1*-driven neoplasms [29, 30]. There are case reports describing very prolonged responses of *BRCA2*-mutated tumors to melphalan [31, 32].

## PARP inhibitors

There are a few PARP inhibitors (PARPi) approved for the clinical use. The guidelines for administration of PARPi are complicated, as the registration trials involved different categories of patients. Early clinical investigations included mainly patients with germ-line *BRCA1/2* mutations [33]. Subsequent studies also considered other categories of tumors with evidences of homologous recombination deficiency (HRD), e.g., cancers with somatic inactivation of *BRCA1/2*, or tumors driven by mutations in other genes of DNA repair pathways, or neoplasms characterized by high-level chromosomal instability. In addition, several trials relied on the fact that the majority of OCs display so-called BRCAness phenotype, i.e. they are characterized by some degree of HRD, and, consequently, platinum sensitivity; therefore, the use of PARPi maintenance therapy in platinum-sensitive OC patients could be considered irrespective of laboratory assays. In fact, these studies utilized phenotypic criteria for the selection of the patients, i.e. the mere fact of the response to platinum agents was taken as a chance to obtain further benefit from PARPi [34]. For the time being, talazoparib is the only PARPi, whose indications are limited to *BRCA1/2* germ-line mutation carriers, with BC being an approved indication [35]. Niraparib has been registered only for OC patients as the maintenance therapy for the platinum-responsive disease and as a single-agent treatment for *BRCA1/2*-driven or chromosomally-unstable OC after exposure to multiple lines of therapy [36, 37]. Similar indications for OC treatment are formulated for rucaparib; rucaparib is also recommended for pretreated prostate cancers carrying germ-line or somatic *BRCA1/2* mutations [38, 39]. There are several scenarios for the use of olaparib as the maintenance therapy in OC, which include both *BRCA1/2*-associated and non-selected platinum-responsive OCs [40–42]. Use of olaparib in BC patients is limited to *BRCA1/2* germ-line mutation carriers [43]. Olaparib is also recommended as a maintenance treatment for hereditary *BRCA1/2*-driven pancreatic cancer [44]. Guidelines for the use of olaparib in castrate-resistant prostate cancer are based on the presence of germ-line or somatic mutations in several genes involved in DNA repair by homologous recombination [45]. There are multiple recently completed or ongoing PARPi trials; therefore, the list of PARPi and the spectrum of approved indications are likely to expand in the near future. It is anticipated, that PARPi will be increasingly used for the treatment of *BRCA1/2*-like sporadic tumors and that adjuvant PARPi regimens will enter clinical practice [46–48].

The invention of PARPi resulted in significant changes in the landscape of cancer treatment. It is beyond the doubt that the use of PARPi is associated with medically relevant improvement of disease outcomes, although at least some real-world studies produce more modest estimates as compared to the registration trials [49]. There are several issues requiring consideration. The mechanisms of action of PARPi demonstrates significant overlap with platinum agents, i.e. these two categories of drugs are seemingly interchangeable in some circumstances [50]. Cisplatin and carboplatin have significant adverse effects and several contraindications, which are not applicable to PARPi, therefore PARP inhibitors are certainly the choice for patients with poor tolerability to platinum compounds [50–52]. However, it is of concern that the available trials usually did not consider the direct comparison between PARPi and cisplatin/carboplatin, while some of them certainly could [53]. For example, the success of adjuvant PARPi trial [48] suggests that evaluation of adjuvant platinum-based therapy is also feasible in BC patients carrying *BRCA1/2* germ-line mutation. PARPi are expensive, hence their comparative assessment towards other drugs has not only medical but also economical relevance [54, 55]. The trend of extending of PARPi indications beyond the tumors driven by germ-line *BRCA1/2* mutations needs to be followed [40, 41, 56, 57]. There are data suggesting that the best responders to PARPi and platinum compounds are accumulated mainly within patients with *BRCA1/2*-driven hereditary cancers, while sporadic tumors with evidences for BRCAness/HRD phenotype often demonstrate less pronounced although still medically meaningful response to *BRCA1/2*-specific therapy [4, 42, 58]. The in-depth analysis of prostate cancer patients receiving olaparib revealed that mainly *BRCA1/2* mutations were associated with the tumor response, while subjects with alterations in other genes of HRD pathway derived no benefit from this drug, despite that the registration documents pooled together *BRCA1/2* and non-*BRCA1/2* mutations [59, 60]. Furthermore, there are data suggesting that only *BRCA2* but not *BRCA1* mutations are associated with high efficacy of PARPi in prostate cancer [61]. Use of PARPi usually results in the restoration of DNA repair, often by the secondary mutation in *BRCA1/2* genes [28]. These tumors are almost certainly cross-resistant to platinum-based therapy and some other cytotoxic drugs. Surprisingly, there is relatively little discussion in the medical literature regarding the remaining treatment options after the failure of PARPi; some clinical data indicate that the use PARPi may expectedly compromise the efficacy of subsequent therapeutic regimens [62].

## Immune therapy

*BRCA1/2*-driven tumors exhibit chromosomal instability, which results in accumulation of genomic rearrangements and, possibly, emergence of cancer-specific antigens [63]. Preclinical studies confirmed increased antigenicity of *BRCA1*-associated tumors and demonstrated that the use of inhibitors of immune checkpoints is a promising treatment option [64]. Matsuo et al. [65] utilized nivolumab in 6 heavily pretreated patients with *BRCA1/2*-related ovarian cancer and observed objective tumor responses in 4 cases. There are data suggesting that platinum and PARPi treatment may increase immunogenicity of tumor cells [66–70]. Multiple clinical trials involving a combined use of PARPi and immunomodulatory agents are currently underway, and the first results suggest a promise of this approach for *BRCA1/2*-associated malignancies [58, 68, 71, 72]. The analysis of already existing data sets is also of potential importance. For example, combination of atezolizumab with nab-paclitaxel is now routinely utilized for the first-line treatment of metastatic triple-negative BC if the tumor-infiltrating immune cells express PD-L1 [73]. A significant portion of women with triple-negative BC carry germ-line *BRCA1* mutations [74]; it would be of interest to evaluate the efficacy of the above doublet in this subset of patients and to compare the efficacy of immune therapy with the outcomes of platinum-based treatment.

## Loss-of-heterozygosity testing and other supporting laboratory assays

Tumor-selective loss of the remaining *BRCA1/2* allele is a key event determining antitumor activity of platinum compounds and PARP inhibitors. It is essential to recognize that the mere presence of *BRCA1/2* germ-line mutation in the genome of a given cancer patient is not always a reliable indication for the administration of the above drugs. There are some tumor types besides breast and ovarian cancer whose risk is somewhat elevated in *BRCA1/2* mutation carriers, however these tumors do not always display loss-of-heterozygosity (LOH) for *BRCA1/2* locus, i.e. they retain the wild-type *BRCA1/2* allele [75]. Furthermore, even breast and ovarian carcinomas arising in *BRCA1/2* mutation carriers do not always have LOH at the *BRCA1/2* locus, and, expectedly, tumors with retention of the normal *BRCA1/2* gene copy show limited sensitivity to platinum drugs [76]. This heterogeneity is currently not considered in daily clinical practice, e.g., *BRCA1/2* LOH or HRD testing is not incorporated in the decision-making process for patients with hereditary cancers. It is likely, that in some future the process of drug choice will be supported by the additional analysis of tumor genome. *BRCA1/2*-driven tumors have characteristic “genomic scars”, which are caused by chromosomal instability. These BRCAness genetic profiles can be reliably determined by the next generation sequencing (NGS). NGS technologies are gradually becoming more cost-effective and user-friendly, and the same applies to the BRCAness assays. Recent studies suggested some simplified approaches for the analysis of BRCAness (HRD) phenotype, which appear to be suitable for routine clinical use [4, 56, 57].

## Acquired resistance to *BRCA1/2*-specifc therapy

Clinical analysis of novel drugs or treatment regimens presents an ethical challenge, especially for tumors which are more or less responsive to standard therapeutic schemes. Consequently, early-phase clinical trials usually involve heavily pretreated patients, or, alternatively, add a novel drug to the standard therapy backbone. Cancers arising in *BRCA1/2* mutations carriers constitute an especial category of malignancies, as they critically change their biological properties over the course of treatment. The analysis of tumors exposed to platinum therapy or PARPi revealed instances of the rescue of BRCA1/2 function, which is achieved by the second mutation in the affected gene, and, consequently, by the restoration of *BRCA1/2* open reading frame [24, 28]. These data are supported by clinical observations of extraordinarily good response in patients with deletion of large fragments of BRCA1/2 genes, i.e., in instances where BRCA1/2 function cannot be restored by the secondary mutation [51, 77]. Studies on *BRCA1*-mutated ovarian carcinomas demonstrated the persistence of a small fraction of BRCA1-proficient cells even in chemonaive tumors; these cells rapidly repopulate tumor mass during the first weeks of platinum-based therapy thus explaining the phenomenon of inevitable emergence of platinum-resistance [78, 79]. Therefore, it is potentially error-prone to evaluate the efficacy of *BRCA1/2*-specific therapies in the pretreated patients, as the tumors rapidly lose their target and, therefore, adapt to the pressure of platinum compounds or PARPi [80–82].

Most of patients with advanced cancer present with multiple metastatic foci. While the core genetic events underlying natural cancer development are usually identical in primary tumors and metastatic lumps, the mechanisms of adaptation of each individual metastatic clone to a therapeutic pressure may be more or less unique. Furthermore, when the systemic treatment is indeed highly efficient, the pattern of disease progression is often limited to an expansion of a single tumor lump. Topical radiological or surgical ablation may be considered for the management of patients with oligometastatic disease, and the latter pattern of tumor appearance is characteristic for *BRCA1/2*-driven tumors exposed to platinum-based therapy [83, 84]. In agreement with these data, neoadjuvant chemotherapy, being clearly inferior to primary surgical debulking in non-selected OC patients, provides equivalent survival outcomes in women with highly chemosensitive hereditary ovarian tumors [84, 85].

Intensification of therapy, i.e. the use of drug combination or increased drug doses is a common approach to combat the tumor plasticity. This attitude is applied to potentially curable cancers, for example, to germ-cell tumors and some hematological malignancies. High-dose chemotherapy, being a life-threatening, highly afflictive and very expensive intervention, was utilized as an investigational treatment to patients with metastatic breast cancer some years ago. The analysis of long-term survivors revealed that high-dose chemotherapy is not a preferable option for non-selected BC cases, but may result in very prolonged responses and possibly even cure from the metastatic BC disease in carriers of germ-line *BRCA1/2* mutations [86–88].

## Conclusions

The discovery of hereditary breast-ovarian cancer genes was initially viewed as an advance in preventive medicine, with the focus on timely cancer diagnosis and prophylactic surgery applied to *BRCA1/2* mutation carriers. Studies on molecular pathogenesis of *BRCA1/2*-driven tumors revealed their specific vulnerabilities and shaped the concept of synthetic lethality [89]. While the actual clinical efficacy of diagnostic screening in *BRCA1/2* heterozygotes turned out to be lower than initially foreseen [90], we are witnessing a spectacular breakthrough in systemic treatment of *BRCA1/2*-associated tumors. Current guidelines already consider *BRCA1/2* testing for the adjustment of therapeutic schemes in patients with breast, ovarian, prostate and pancreatic malignancies. Lessons learned from hereditary cancers led to the extension of many drug indications to sporadic tumors carrying BRCAness phenotype. *BRCA1/2* testing and related laboratory procedures are currently guiding many therapeutic decisions. The full-scale *BRCA1/2* analysis or HRD evaluation require significant time and resources, so they are still poorly compatible with the choice of neoadjuvant or first-line therapy. However, given that the acquisition of tumor resistance to systemic treatment often involves restoration of BRCA1/2 function, it is essential to ensure that patients with *BRCA1/2*-driven tumors receive *BRCA1/2*-specific drugs (e.g., platinum-based regimens) in the very beginning of therapeutic intervention. Significant reduction of the turn-around-time for multigene assays is a critical need for further advances in molecular cancer medicine.

## Data Availability

Not applicable.

## Abbreviations

BC: Breast cancer
HRD: Homologous recombination deficiency
NGS: Next generation sequencing
OC: Ovarian cancer
PARPi: Poly (ADP-ribose) polymerase inhibitors
pCR: Pathologic complete response
